# Successful treatment of recurrent immunoglobulin a nephropathy using steroid pulse therapy plus tonsillectomy 10 years after kidney transplantation: a case presentation

**DOI:** 10.1186/s12882-018-0858-9

**Published:** 2018-03-14

**Authors:** Haruki Katsumata, Izumi Yamamoto, Yo Komatsuzaki, Mayuko Kawabe, Yusuke Okabayashi, Takafumi Yamakawa, Ai Katsuma, Yasuyuki Nakada, Akimitsu Kobayashi, Yudo Tanno, Jun Miki, Hiroki Yamada, Ichiro Ohkido, Nobuo Tsuboi, Hiroyasu Yamamoto, Takashi Yokoo

**Affiliations:** 1 Division of Nephrology and Hypertension, Department of Internal Medicine, The Jikei University School of Medicine, 3-25-8, Nishi-Shimbashi, Minato-ku, Tokyo, 105-8461 Japan; 2 Department of Urology, The Jikei University School of Medicine, Tokyo, Japan; 3 Department of internal Medicine, Atsugi City Hospital, Kanagawa, Japan

**Keywords:** IgA nephropathy, Kidney transplantation, Tonsillectomy, Steroid, Calcineurin inhibitor nephrotoxicity, Case report

## Abstract

**Background:**

Both prevention and treatment of recurrent immunoglobulin A nephropathy (IgAN) in kidney transplant recipients are important since recurrent IgAN seems to affect long-term graft survival. We present here a case of recurrent IgAN that was successfully treated using steroid pulse therapy plus tonsillectomy 10 years after kidney transplantation.

**Case presentation:**

A 46-year-old male was admitted for an episode biopsy with a serum creatinine level of 1.8 mg/dl and proteinuria (0.7 g/day). Histological features showed recurrent IgAN (only focal segmental mesangial proliferation) and severe arteriolar hyalinosis partly associated with calcineurin inhibitor toxicity, with limited interstitial fibrosis and tubular atrophy (5%) (IF/TA) 8 years after transplantation. Sodium restriction and conversion from cyclosporine to tacrolimus successfully reduced his proteinuria to the level of 0.15 g/day. However, 2 years later, his proteinuria increased again (1.0 g/day) and a second episode biopsy showed global mesangial proliferation with glomerular endocapillary and extracapillary proliferation accompanied by progressive IF/TA (20%). The steroid pulse therapy plus tonsillectomy successfully decreased his proteinuria and he achieved clinical remission 3 years after this treatment.

**Conclusion:**

This case, presented with a review of relevant literature, demonstrates the difficulty and importance of the treatment of recurrent IgAN and calcineurin inhibitor arteriolopathy, especially in long-term kidney allograft management.

## Background

Immunoglobulin A nephropathy (IgAN) is a common recurrent glomerulonephritis that affects transplanted kidney allograft survival. The rate of allograft loss at 10 years is similar with IgAN and other glomerulonephritis [1]. However, a recent study showed that graft survival in patients with IgAN has gradually worsened and death-censored graft survival at 15 years was approximately 10% lower compared with other glomerulonephritis [2]. Steroid pulse therapy is the standard treatments for recurrent IgAN, but difficult to manage, especially in long-term kidney allograft. We here report a case of IgAN that reoccurred 10 years after kidney transplantation, which was successfully treated. In addition, this case showed severe arteriolar hyalinosis partly induced by long-term use of calcineurin inhibitors (CNIs). CNI arteriolopathy affects allograft survival; some investigators have tried to withdraw or avoid these drugs, as their use is now controversial. We also review this important problem.

## Case presentation

**Fig. 1:**
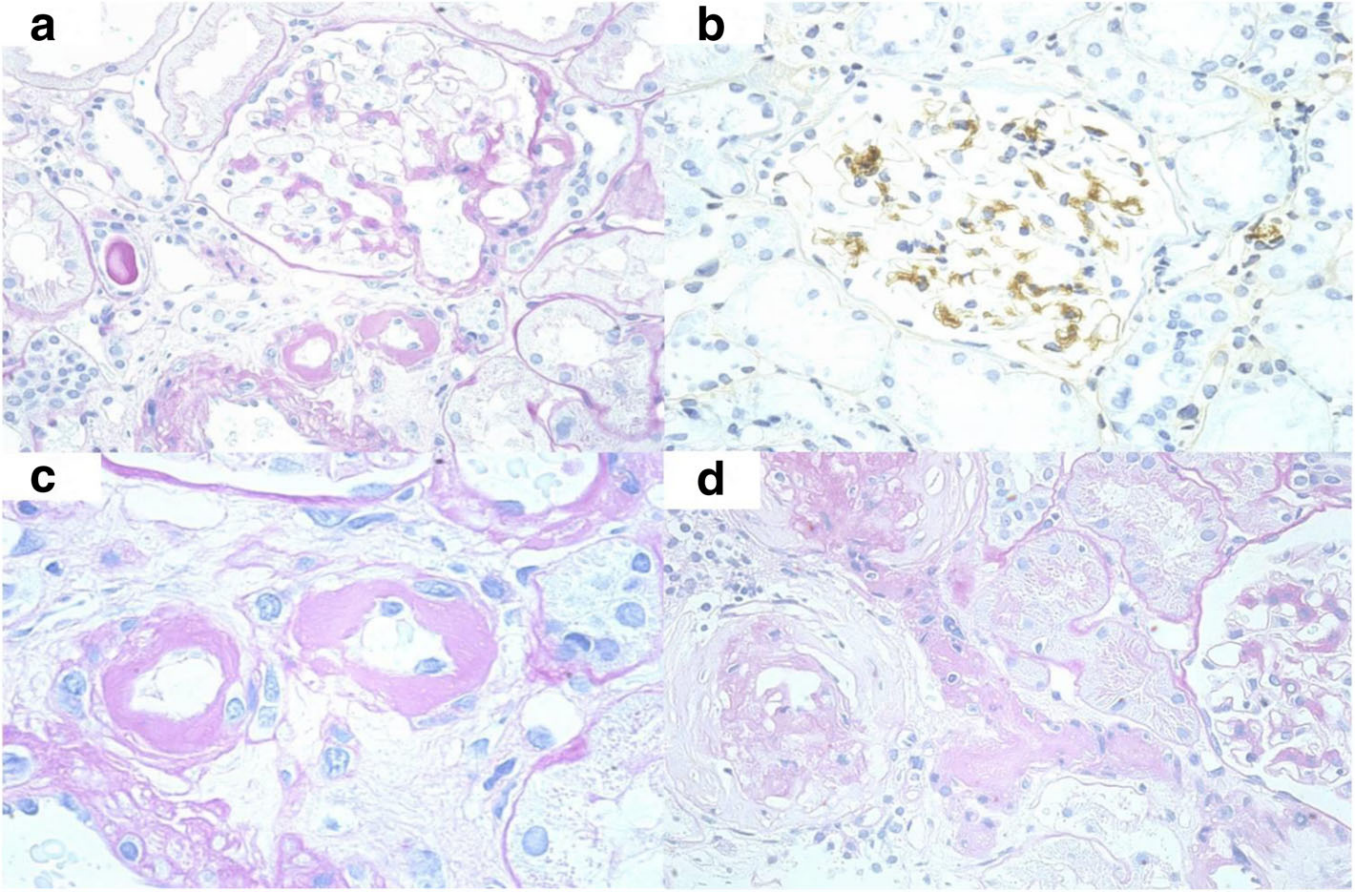
First episode biopsy was performed 8 years after transplantation. Light microscopy showed no evidence of rejection. (**a**) A segmental mesangial hypercellularity can be seen. (**b**) An immunofluorescence study showed mild-to-moderate positivity in the mesangial area for IgA. Positivity for IgM and C3 was also seen (not shown). (**c**) An arteriolar hyalinosis caused by CNI nephrotoxicity was remarkable. (**d**)Two branches of severe arteriolar hyalinosis seemed to develop global glomerular sclerosis

A 46-year-old Japanese man was admitted to our hospital for an episode kidney allograft biopsy because his proteinuria and serum creatinine had increased at the level of 0.7 g/day and 1.8 mg/dl, respectively. His first kidney biopsy was performed at 19 years of age for proteinuria and hematuria (this biopsy result could not be obtained). However, he had not received any treatment for his kidney disease. After 16 years, he presented at our hospital with a visual loss caused by hypertensive retinopathy, and blood examination showed end-stage renal disease. After undergoing peritoneal dialysis for 1.5 years, he received a living-related kidney transplantation from his mother when he was 36 years old. ABO blood types were compatible. The immunosuppressive therapy consisted of tacrolimus, mycophenolate mofetil, methylprednisolone, and basiliximab. The allograft demonstrated excellent early function, with a serum creatinine (S-Cr) level and proteinuria of 1.5 mg/dl and 0.2–0.3 g/day, respectively. The 1-month and 1-year protocol biopsy showed neither evidence of rejection nor recurrence of primary disease. However, he developed pure red cell aplasia caused by parvovirus; therefore, tacrolimus was converted to cyclosporine and he recovered from this infection immediately. His S-Cr levels increased to 1.8 mg/dl, with the appearance of proteinuria (0.7 g/day) 8 years post transplantation. A first episode biopsy was performed and histopathology showed no evidence of rejection with limited interstitial fibrosis and tubular atrophy (5%). However, global sclerosis was observed in 15 out of 36 glomeruli (42%). The rest of the conserved glomeruli showed focal segmental mesangial proliferation with IgA deposition. Of note, severe arteriolar hyalinosis partly associated with CNI arteriolopathy was evident (Banff 2013 classification: t0, i0, g0, v0, ptc0, ct1, ci0, cg0, cv0, aah3). He was diagnosed with recurrence of IgAN (Oxford classification: M1, S1, E0, T0) and severe arteriolar hyalinosis. We estimated that the patient had no active lesion due to IgAN because there was neither endocapillary nor extracapillary proliferation. On the other hand, 42% of global sclerosis seems to be led by severe arteriolar hyalinosis (Fig. 1). Because he had already taken angiotensin II receptor blocker, we did not administer further drugs. Dietary sodium restriction and conversion of cyclosporine to tacrolimus reduced urinary protein to the level of 0.15 g/day.

**Fig. 2:**
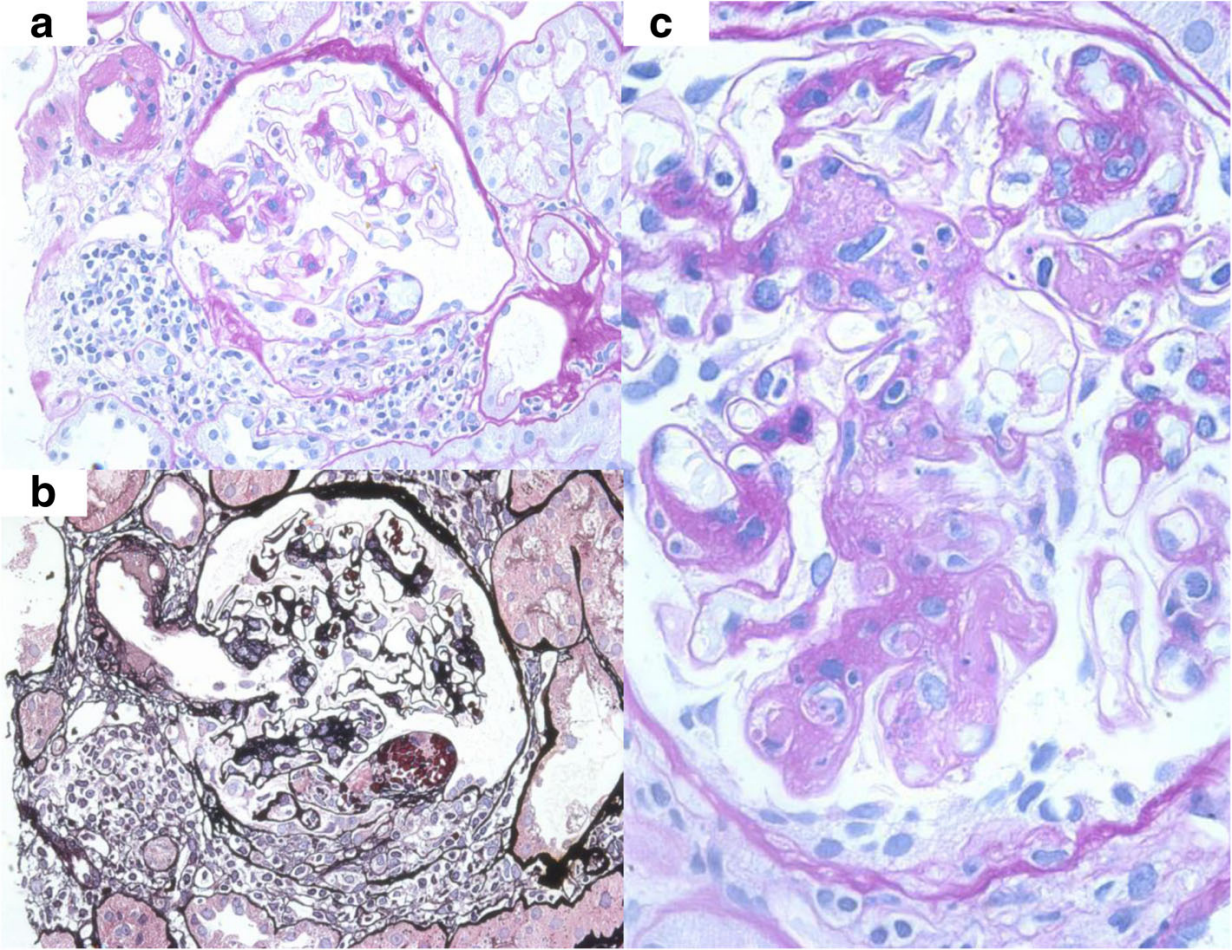
Second episode biopsy that was performed 10 years after transplantation also showed no evidence of rejection. (**a**), (**b**) Extracapillary proliferations were observed in 2 of 19 glomeruli. (**c**) A global mesangial proliferations and endocapillary proliferations were seen

**Fig. 3:**
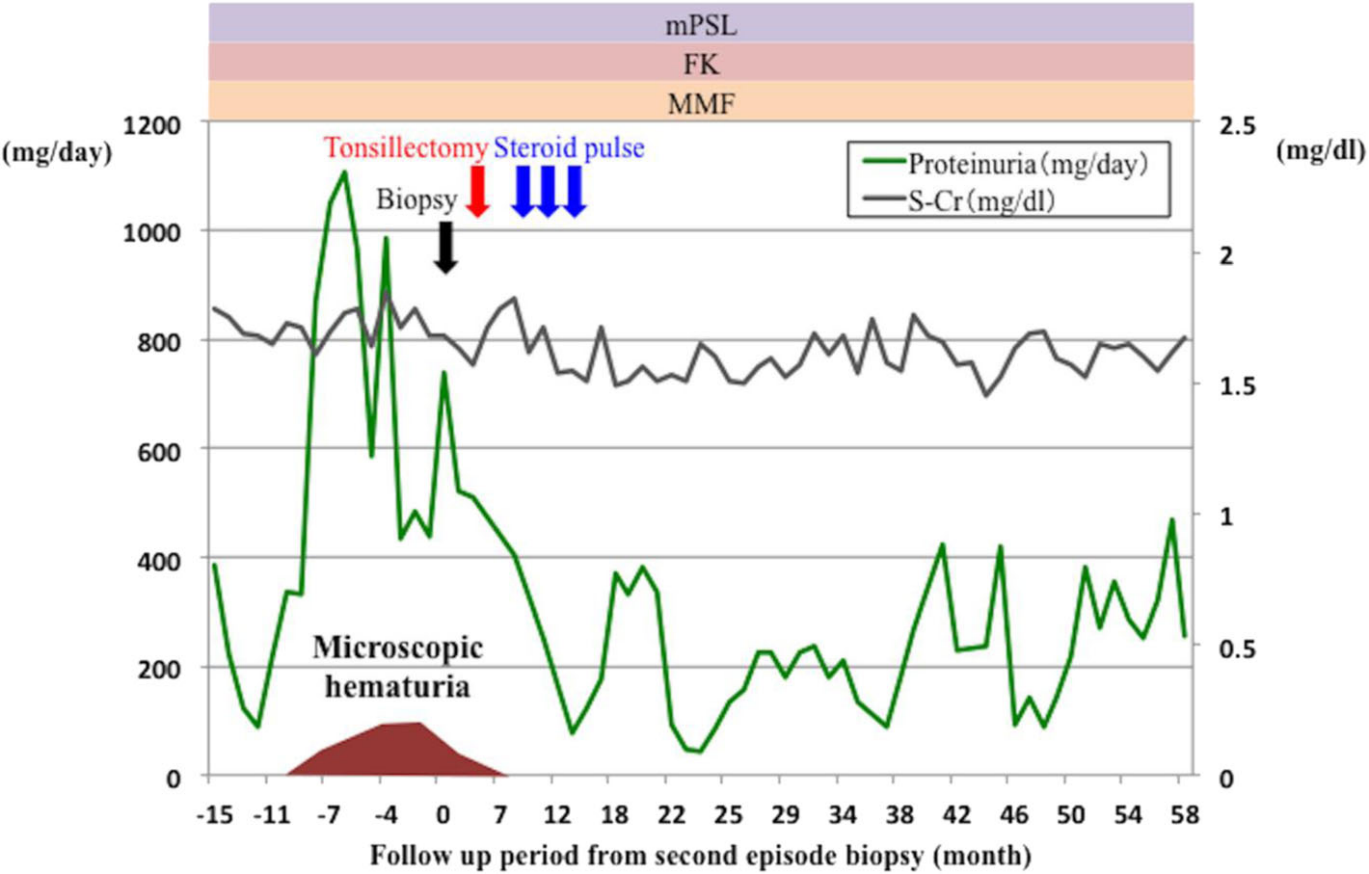
Patient clinical course. His proteinuria gradually decreased and was maintained around 300 mg/day for 3 years after the therapy. mPSL methyl prednisolone; FK tacrolimus; MMF Mycophenolate mofetil

However, 2 years later (10 years after transplantation), he presented proteinuria again at the level of 1.0 g/day accompanied by microscopic hematuria (urinary red blood cell count was 5–9/high-power field). Therefore, a second episode biopsy was performed. Histopathology showed diffuse global mesangial proliferation and mild interstitial fibrosis (20%); moreover, endocapillary and extracapillary hypercellularity was evident in two out of 19 glomeruli (Fig. 2). In the mesangium, immunofluorescence revealed multiple immunoreactivities for IgA, immunoglobulin M (IgM), complement component 3 (C3), and complement component 1, q subcomponent (C1q). We diagnosed recurrence of IgA nephropathy again and the Oxford classification showed a higher score (M1, S1, E1, T1) than in the first episode biopsy. This recurrence had happened despite taking immunosuppressive drugs and histologically worsened than first time recurrence after kidney transplantation. These findings predicted disease progression and poor prognosis. Therefore, we considered steroid pulse therapy plus tonsillectomy was more effective in this case than steroid pulse therapy alone. He received tonsillectomy first due to his work’s schedule (He could hospitalize only at this time). Because steroid pulse therapy immediately after tonsillectomy had been reported to increase the bleeding episodes, he received steroid pulse therapy (methylprednisolone 500 mg/day × 3 days × 3 times) few months later. After the treatment, his proteinuria gradually decreased and was maintained around 0.3 g/day for 3 years after started therapy (Fig. 3).

## Discussion

Recurrent glomerulonephritis is an important problem affecting transplanted kidney allograft survival. A previous report showed no significant differences in allograft loss at 10 years with IgAN and other glomerulonephritis [1]. However, Moroni et al. demonstrated that graft survival in patients with IgAN had become gradually worse and death-censored graft survival at 15 years was approximately 10% lower compared with that in controls (62.6% vs 72.4%) [2]. Furthermore, in this retrospective study, IgAN recurred in 22.1% of patients and these patients had a worse prognosis compared with non-recurrent IgAN patients; therefore, poor outcomes in IgAN patients are likely to be caused by its recurrence. Recurrence is an important cause of allograft loss in other glomerulonephritis as in IgAN, but IgAN especially worsened as noted in long-term observation [3]. Thus, aggressive medical treatment for recurrent IgAN in long-term transplant recipients is required to improve graft outcomes.

In native kidney biopsies, endocapillary proliferation is known to be a risk factor for the progression of IgAN [4]. In addition, a previous study that evaluated the outcome in recurrent IgAN after transplantation had demonstrated proteinuria (more than 1 g/day), elevation of S-Cr level, and pathological findings of more than 30% glomerulosclerosis and interstitial fibrosis were the risk factor of disease progression [5, 6]. In our case, first episode biopsy showed increased S-Cr level from 1.5 to 1.8 mg/dl and histopathology showed 42%(≻30%)glomeruloscrlerosis. In line with such reports, our case showed disease progression for the 2 years after the first episode biopsy. However, we did not treat for recurrent IgAN because the disease activity was not clearly evident at first biopsy.

Interventional solutions for recurrent IgAN in kidney transplant recipients have not been sufficiently studied; therefore, these patients should be treated similarly as non-transplant IgAN patients at present. However, kidney transplant recipients have already taken multiple immunosuppressants and hence recurrent IgAN often becomes more difficult to treat, so we performed tonsillectomies in addition to steroid pulse therapy. Various methods of steroid pulse therapy based on dose, dosing period, and interval have been proposed at present. Our method was based on the protocol that Pozzi et al. had reported previously [7]. Although it is still controversial whether tonsillectomy should be performed in IgAN, we consider that tonsillectomy combined with steroid pulse therapy is the better treatment of IgAN for several reasons. First, tonsillectomy has been reported to be safe surgery whose mortality rate is much low (0.0006%) [8]. Other reports also showed low risk of tonsillectomy (the prevalence rate of bleeding: 1.3–2.9%) [9–15]. Second, tonsillectomy can remove abnormal polymeric IgA1, which is known as the pathogenesis of IgAN [16]. A combined tonsillectomy and steroid pulse therapy is thought to be effective to decrease the production of polymeric IgA1 by both removal of tonsils and suppression of plasma cells. Finally, several studies have clinically revealed the efficacy of combined therapy. The recent prospective randomized-control study showed that in native kidneys, the percentage decrease in proteinuria at 12 months was better in combination with steroid pulse therapy and tonsillectomy compared to that with steroid therapy alone. Although the clinical remission rate was not significantly different between steroid therapy plus tonsillectomy and steroid therapy alone, the combined therapy was the independent factor contributing to the disappearance of proteinuria (odds ratio 2.98) [17]. Similarly, a more recent meta-analysis that included 14 studies (1794 patients) noted that the remission rate of proteinuria was better in the combination therapy (odds ratio 3.15) [18]. Additionally, recent randomized controlled study has examined the effect of steroid pulse therapy combined with tonsillectomy on clinical remission by pathological sub-analysis. This study has suggested that combined therapy has a greater benefit of clinical remission than steroid therapy alone in the moderate to severe pathological case that showed more than 25% of glomeruli exhibiting crescents, segmental sclerosis or global sclerosis (odds ratio 8.17) [19]. Our case also matched these criteria (42.1%: two crescents and 6 global sclerosis was observed in 19 glomeruli). In this context, tonsillectomy in addition to steroid pulse therapy seems beneficial in recurrent IgAN as well. Several case reports and retrospective cohorts showed the proteinuria-reducing effects of tonsillectomy in transplant recipients [20, 21]. In our case, proteinuria was improved after steroid pulse therapy plus tonsillectomy. Therefore, we should consider performing tonsillectomy if the disease is progressive or difficult to treat by other therapy. However, based on the previous report that evaluated the preventive effects of pretransplant tonsillectomy on IgAN recurrence, tonsillectomy may not be useful for preventing IgA recurrence [22].

Another important issue as shown in this case was CNI toxicity. Histopathology showed severe arteriolar hyalinosis partly associated with CNI toxicity. Low dose tacrolimus is less harmful than cyclosporine in both renal and non-renal organ transplantation [23, 24]. Ojo et al. reported that the relative risk associated with cyclosporine was 1.24 [25]. Therefore, we reconverted cyclosporine to tacrolimus after the first episode renal biopsy. CNI withdrawal or avoidance is a well-known risk factor for graft rejection. A recent prospective study that tried to taper the tacrolimus dose in stable transplant recipients became difficult to continue because of high rates of acute rejection (4 of 14 patients) and de novo donor-specific antibody appearance (5 of 14 patients) [26]. Therefore, change from CNI to mammalian target of rapamycin inhibitor (mTORi) may be the alternative treatment. In fact, some investigators reported improvement in glomerular filtration rate after change to mTORi, without any increase in graft rejection [27, 28]. However, a systematic review of randomized control studies showed that the risk of rejection at 1 year is higher in mTORi-based treatment (relative risk 1.72) [29]. Additionally, Gallon L et al. reported that T-cell alloreactivity increased after switching tacrolimus to sirolimus, which indicates that conversion runs the risk of graft rejection [30]. Hence, we did not convert to mTORi in this case.

## Conclusion

We present that steroid pulse therapy plus tonsillectomy is effective even in patients with long-term recurrent IgAN. Managing long-term CNI toxicity is another important issue. Prospective studies are needed to assess the efficacy of such management in cases of long-term kidney allograft.

## Abbreviations

CNI: calcineurin inhibitor
IgAN: immunoglobulin A nephropathy
mTORi: mammalian target of rapamycin inhibitor
